# Debt Relief as a Last Resort for the Lender of Last Resort? Monetary Financing — Doing It Right

**DOI:** 10.1007/s10272-021-0984-7

**Published:** 2021-08-05

**Authors:** Arne Hansen, Dirk Meyer

**Affiliations:** University of the Federal Armed Forces Hamburg, Helmut Schmidt University, Holstenhofweg 85, 22043 Hamburg, Germany

## Abstract

The coronavirus crisis has led to a sharp increase in the debt-to-GDP ratios of the euro area member states. Without external support, access to the capital market could be seriously threatened in the medium term for Italy, but also for other member states. While the Pandemic Emergency Purchase Programme, which is designed as a monetary policy instrument, is regarded by some as a violation of the prohibition of monetary financing, the Next Generation EU recovery fund is likely to direct the fundamental structures of the European Union towards a fiscal union with considerable redistribution elements. This article analyses an alternative strategy, namely debt relief by the European System of Central Banks through an EU debt agency. Such a scheme would be possible without amending the EU treaties and would avoid negative equity at the central banks. The question is under what circumstances would this approach be suitable and proportionate?

The COVID-19 crisis has confronted the European Union (EU) with new challenges. After the special summit of 21 July 2020, member states responded with a financial framework totalling €1,824.3 billion. This framework comprises the multiannual financial framework for 2021-2027 (€1,074.3 billion) and the Next Generation EU (NGEU) recovery instrument (€750 billion). This special budgetary instrument entails elements of a structural departure from traditional EU law (Meyer, 2021). Created on the basis of the EU civil protection legislation clause (Article 122 of the Treaty on the Functioning of the European Union, TFEU), it covers not only the euro area but all EU member states. The aid will not be subject to conditions or in-depth checks. For the first time, the EU will be taking out substantial loans of more than €750 billion. In order to finance these loans on its own, the EU will be given independent fiscal sovereignty. If individual member states fail to repay their EU loans, the other member states will guarantee these loans proportionately – Eurobonds, albeit under a different name.

In this article, we analyse a potential alternative to the €750 billion recovery programme on the basis of proportionately balanced debt relief for the euro area members and EU member states. We also present a further debt relief scenario.[1] The aim is a limited reduction of the debt in crisis-hit countries, in particular so as to restore their debt sustainability or at least to ensure that it is no longer threatened. Another aim is to avoid a redistribution element and a transfer of powers to the EU level. At the same time, this debt relief must conform to the provisions of the Treaty on European Union (TEU) and the TFEU.

The recovery fund is mainly designed to help the heavily indebted euro area member states. In the past, some of these countries have violated EU debt regulations and for this reason would be likely to lose their access to the capital market if they had to secure suitable loans on their own. For Greece, Italy and Portugal, the debt-to-GDP ratios at the end of 2021 were forecast to be 208.8%, 159.8% and 127.2% respectively (European Commission, 2021a). With this new "EU umbrella", it will be possible to avert impending sovereign default, accommodate Italy's request to circumvent the European Stability Mechanism (ESM) with conditions-based aid and avoid having to in-

1 For alternative scenarios, see Hansen and Meyer (2020a, 291-303).

crease the ESM's funds as would otherwise be necessary. Moreover, with its Pandemic Emergency Purchase Programme (PEPP), which is passed off as a monetary policy instrument (European Central Bank, 2020a), the European System of Central Banks (ESCB) will in effect assume the role of a lender of last resort for countries.[2] This raises legal concerns, particularly in light of the German Constitutional Court's ruling on Public Sector Purchase Programme (PSPP, European Central Bank, 2020b) government bond purchases (BVerfG 2 BvR 859/15).[3] So what could be a more obvious political and economic measure than a debt relief on the basis of public sector securities purchased by the ESCB as part of the PSPP and the PEPP?[4] This would also enable the member states to establish appropriate national pandemic programmes without going through the EU budget. These programmes would be financed by national taxes (e.g. a one-off capital levy) or new government loans. This would avoid the problems of the recovery programme and its regulatory consequences as well as the legally questionable purchases of government bonds by the ESCB, which may not be necessary then.[5]

The present article offers an observation of the legal aspects of the considered ESCB debt relief, followed by an analysis of options for conducting such debt relief. After a discussion of the possible economic consequences, two detailed numerical scenarios are presented and their implications are scrutinised.

## Legal aspects

The President of the European Parliament, David Sassoli, stated in November 2020, that debt cancellation would be "an interesting working hypothesis, to be reconciled with the cardinal principle of debt sustainability" (Arnold and Hindley, 2020). Along the same lines, in a letter published by some European newspapers in February 2021, a group of more than 100 economists called on the European Central Bank (ECB) to cancel the government debt that it owns or to convert it into perpetual bonds with 0% interest rate (Andor et al., 2021).[6] The President of the ECB, Christine Lagarde, so far has rejected such ideas because "there is Article 123 of the treaty, which prohibits that kind of approach", and "anything along those lines would simply be a violation of the treaty" (Arnold, 2020). Obviously, the prohibition of monetary financing in Article 123(1) of the TFEU represents a legal hurdle (Deutscher Bundestag, 2021, 8-10); the article prohibits "the purchase directly from them [the member states] by the European Central Bank or national central banks of debt instruments". The potential circumvention of the prohibition of monetary financing was scrutinised not least by the German Constitutional Court (BVerfG 2 BvR 859/15) on 5 May 2020 with regard to the PSPP on the basis of various criteria.[7] This shows that secondary market purchases can also be considered monetary financing and that determining what is legal and what is illegal is not always easy. According to the court, "the decisions on the adoption and implementation of the PSPP ultimately do not amount to a qualified violation of Article 123(1) TFEU given that, based on a proper application of the criteria …, it is not ascertainable that the purchases … manifestly circumvent the prohibition of monetary financing" (BVerfG 2 BvR 859/15, para. 197).[8]

But how, in legal and practical terms, would we manage the hypothetical but by all means possible constellation in which, as matters stand, the debts of a considerable number of member states are no longer sustainable and these states are on the brink of losing their access to the capital market and, in response to this crisis, the ESCB as a lender of last resort would (partly) waive the redemption of government bonds – i.e. it would effectively cancel them at its own expense? In this case, it would be appropriate to speak of a debt relief that, in effect, would simultaneously be a form of sovereign debt monetisation or monetary financing (von Lewinski, 2011, 455).

Given the independence of the ESCB and its primary commitment to maintaining price stability (Article 282 TFEU) and the principle of conferral (Article 5(2) TEU), this procedure would require special legitimation. Using a euro zone emergency as a justification, the legal concept of a temporal constitutional exemption could be ap-

2 On the ECB as a lender of last resort for countries during the coronavirus crisis, see Neyer (2020, 18-19).

3 See the findings of a study by Hansen and Meyer (2020b).

4 See Röhl (2020), whose proposal served as an initial impetus for our work. Quite fittingly, the total volume of ESCB sovereign debt purchases under the PSPP and PEPP is close to the expected increase in euro area debt from 2019 to 2021, see German Council of Economic Experts (2021, para. 114).

5 According to the German Council of Economic Experts (2021, para. 114), the "securities purchases constitute a large-scale support measure for the … financing costs of the member states" so that "even highly-indebted member states continue to be able to fund themselves in the bond market."

6 Andor et al. (2021) argue "that the word 'cancellation' does not appear neither in the treaty nor in the protocol" on the ESCB and that the ECB "can even print money to compensate for these losses".

7 The ruling (BVerfG 2 BvR 859/15) highlights the purchase limit of 33% and the distribution of purchases according to the ECB's capital key (para. 217) as the two decisive criteria for "an overall assessment and appraisal" (para. 215). This prevents market power that influences prices (para. 201-202) and preferential fiscal policy treatment for select member states (para. 203).

8 In contrast, an empirical analysis by the authors comes to different findings. See Hansen and Meyer (2020b).

plied.[9] As a derogation on the same level as the TFEU, an amendment (Article 48 TEU) would be necessary to legitimate a single limited exception by legislation. This would, of course, be politically difficult because, alongside the no-bailout clause, the prohibition of monetary financing is one of the key rules of EU economic law. For this reason as well, it would be advisable to act within the scope of current EU law.

## Options of ESCB debt relief

In the following, we analyse different ways of conducting ESCB debt relief. The options must fulfil all of the following four conditions:

- a (considerable) reduction in the debt-to-GDP ratios in particular of those euro area member states whose debt sustainability is acutely at risk
- avoidance of negative equity at the ECB and national central banks (NCBs)
- avoidance – as much as possible – of the unequal treatment of member states through redistribution
- compatibility with existing EU laws.

### Immediate write-off leads to negative equity

A number of suggestions are based on the purchase of government bonds, which the ECB or the ESCB would convert into perpetual or 100-year bonds on an interest- and redemption-free basis.[10] As a result, the value of the bonds would immediately drop to zero.[11] This leads to the problem of booking the losses resulting from debt relief. The central banks of the ESCB have relatively little equity in relation to their total assets. The ECB has €10.825 billion in equity, with €7.584 billion (81.33%) from the euro area member states, of which €1.999 billion (21.44%) is from the Bundesbank (as of 29 December 2020). The Bundesbank itself has equity amounting to €5.720 billion. Since the debt relief that we are considering amounts to tens to thousands of billions of euros, the associated immediate write-offs – if they were implemented in the near term – would lead to a high level of negative equity at the NCBs and the ECB. It is true that central banks cannot become insolvent because they themselves issue legal tender and can therefore meet any liability by creating money.[12] If they did so, however, they would risk losing trust (damage to their reputation) and political control, while repeating such instances would likely cause inflation.

### ESCB write-off bonds or equalisation claims

Equalisation claims or write-off bonds are one way of writing off government debt by way of debt relief combined with extending the loss period. As a rule, equalisation claims serve as a counter entry – mainly on the balance sheets of banks – if an equity gap arises as a result of political interventions. While equalisation claims must be paid off, write-off bonds are written off over a certain – usually long – period, resulting in a debit for the holder and a credit for the issuer. However, equalisation claims also depreciate with inflation and long redemption-free terms; as a result, the debit/credit effects are similar.[13]

The ESCB write-off bonds considered here are interest-free bonds with a variable annual write-off amounting to the central bank's net profit for the year, which would have otherwise accumulated and would have had to be shown on the balance sheet. They thus cannot be traded on the open capital market. The state's debt decreases annually by the amount of the write-off. The financial burden this places on the central bank is passed on to the taxpayer because no profits are transferred to the state budget. Instead of transferring all or large proportions of the net profit for the financial year to the state budget, as is normally the case, the state's debt is reduced earlier.[14] This is possible with a European debt agency. This EU debt agency would have two functions:

- issue the write-off bonds, thus uncoupling ESCB debt relief from state budgets
- act as a buffer, avoiding negative equity at the central banks by way of write-offs over time while immediately reducing state debt.

9 For more information on national state emergencies, see von Lewinski (2011). Von Lewinski says a financial emergency is only a national emergency if essential state functions can no longer be performed as a result.

10 See, e.g. Pâris and Wyplosz (2014) and Stelter (2020, 153-160). Röhl (2020, 2) writes about waiving the repayment of government bonds bought by the ESCB.

11 Depending on what approach is taken, 100% of the state debt would technically continue to exist, which means that the debt-to-GDP ratios of the various countries would not be reduced.

12 See German Council of Economic Experts (2018, para. 393-396), on the debate about whether and how central banks with negative equity can continue working.

13 In the past, equalisation claims were used during the German currency reforms of 1948 and 1990. To balance out their losses resulting from an asymmetric conversion of claims and liabilities, long-term equalisation claims against the federal government were transferred to the affected commercial banks. See Deutsche Bundesbank (1995, 1996) and Vogelsang (2011).

14 Immediate and complete debt relief is different from Stelter's approach, which envisages an extension of the relief. See Stelter (2020, 156).

Figure 1
**ESCB debt relief: Write-off bonds and relief agreement via EU debt agency**

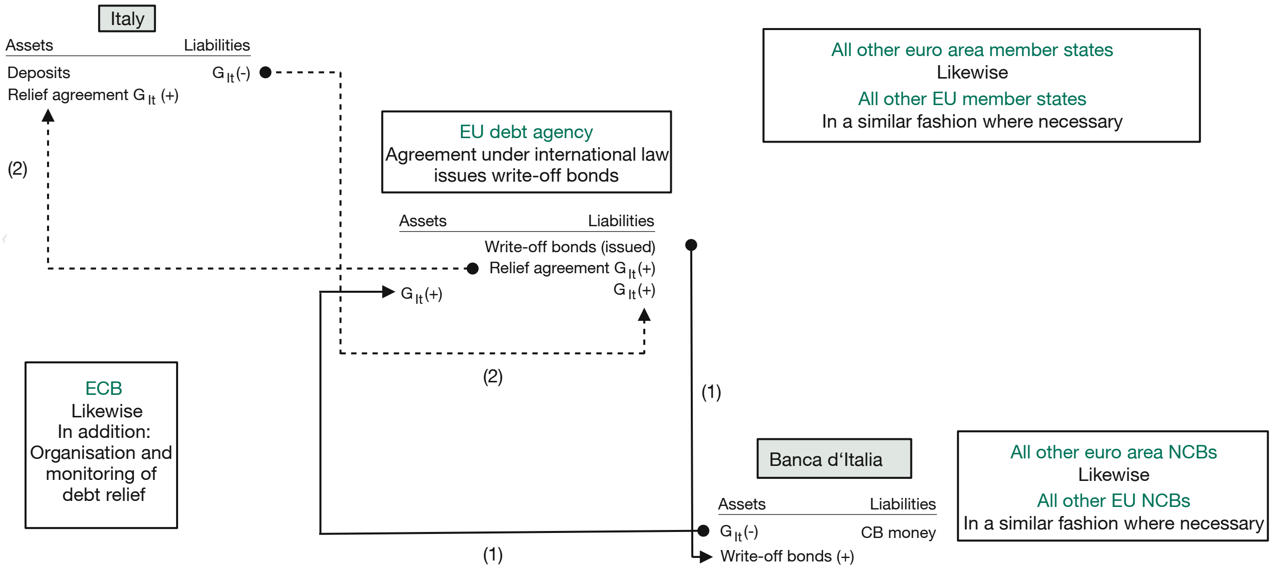

Note: $G_{It}$ stands for government debt securities, Italy; CB stands for central bank.

Source: Authors' own compilation.

The EU debt agency would be responsible for transforming debt relief. Like the ESM, it would be founded as an international, independent financial institution on the basis of a treaty between the euro area member states. It would therefore be legally independent from the EU. Equipped with a banking licence, the EU debt agency could come under the exemption clause of Article 123 TFEU. According to Article 123(1) TFEU, the direct purchase of government bonds by the European Central Bank or national central banks is prohibited. However, these provisions do not apply to "publicly owned credit institutions which, in the context of the supply of reserves by central banks, shall be given the same treatment by national central banks and the European Central Bank as private credit institutions" (Article 123(2) TFEU).[15] Whether the establishment of an EU debt agency with a banking license fulfils these conditions is likely to be controversial. Alternatively, and for reasons of legal security, a selective and temporal constitutional exemption could be legitimised on the basis of Article 48 TEU.

The described approach is shown in Figure 1, with Italy as an example. In step (1), the EU debt agency issues write-off bonds (balance sheet expansion) that are exchanged by the Banca d'Italia for Italian government bonds ($G_{It}$) held there (accounting exchange on the assets side). In step (2), the EU debt agency takes on the government debt ($G_{It}$) in its balance sheet by way of a relief or revocation agreement. Italy thus receives immediate debt relief, which, on the balance sheet, is reflected by an increase in the value of assets and a drop in the debt-to-GDP ratio. At the same time, the EU debt agency can balance out the government bonds ($G_{It}$) that were exchanged by the Banca d'Italia for write-off bonds in step (1). On the assets side there is a gap amounting to the commitments from the write-off bonds and the relief agreement; this excess debt is reduced to zero over the long term. Officially, the EU debt agency functions here as an outsourced subsidiary budget for all euro area countries, which solves the problem of temporally incongruent demands of "immediate relief of government debt" and "avoidance of negative central bank equity".

The same approach is used by the other 18 euro area member countries and the ECB, which convert their (partial stock) holdings of national government bonds into write-off bonds. The ECB would also be responsible for organising and supervising the debt relief process. All EU member states that have not yet introduced the euro are free to reduce their debt accordingly on the basis of their

15 Article 55(c)(ii) of Guideline (EU) 2015/510 explains this in more detail. According to this article, approved business partners must, for example, fulfil the following condition: They must be "publicly owned credit institutions, within the meaning of Article 123(2) of the Treaty, subject to supervision of a standard comparable to supervision by competent authorities under Directive 2013/36/EU and Regulation (EU) No 575/2013".

national currency, independently of the euro area. With this approach, we do not differentiate between the write-off bonds of the various countries. However, there are differences between the substituted government bonds of the euro area member states in terms of their average duration and risk-differentiated average interest rates. The annual write-off rates are also likely to differ because the profits of the individual NCBs will not be the same.[16] From the perspective of distributional neutrality, it could be particularly important to adopt an appropriate interest rate with regard to the write-off bonds. However, the interest accruing at the NCBs would increase their annual surplus and, in turn, the write-off rate in favour of the depreciation of the write-off bonds ("left-pocket-to-right-pocket transaction"). The approach presented here would only lead to the already existing distribution effects that result from the rules on the allocation of the NCBs' monetary income (Article 32, ESCB Statute) and the distribution of the ECB's profits (Article 33, ESCB Statute; Hansen and Meyer, 2020b, 41-42).

### Economic aspects of monetisation

ESCB debt relief leads to capital growth in the accounts of the euro area member countries (liabilities are cancelled), while the NCBs and the ECB record a corresponding loss (assets are cancelled), possibly over long periods of time. If we assume that debt relief is based on the ECB's capital key (see scenario (i) below), redistribution is avoided, except for the waiving of interest, which has an equalising effect. Within a consolidated state-NCB balance sheet (broad scope of consolidation), the items balance each other out, and the change in capital is zero.[17] This distinguishes monetisation through a central bank from a general "haircut" on government debt, which is conducted via the entire bond issue. The latter would also affect holdings in the hands of private entities, which means that the state would experience capital growth on the same scale. There is thus likely to be less resistance to monetisation.

The question arises as to whether any costs at all would be incurred and if so, who would bear them. The loans to the member states provided them with purchasing power to buy domestic goods and services as well as imports. If we assume that, alternatively, a tax had been imposed, this would have involved costs for the taxpayer and there would have been resistance against such a tax. If this is not the case, the increase in public demand could have led to a crowding out of private demand. However, there is no (additional) inflationary effect because the ESCB has already increased the base money supply when it purchased the bonds.

Two fundamental objections remain, however: the issue of sterilisation and that of non-recurrence. At least potentially, a central bank has the option of sterilising the central bank money created with bond purchases through early sales or redemption at final maturity. The non-marketability of write-off bonds cancels this option. Thus, the higher the debt relief, the more severely monetary policy control is impaired.[18] However, another question is whether the capital market in particular would be receptive to bonds of heavily indebted euro area countries. In general, a reduction of the ongoing asset purchase programmes appears problematic at present and will remain so in the foreseeable future.[19] After all, since the beginning of the government bond purchase programmes in 2010, the – mostly long-term – securities were held until their final maturity and the redemption sums were reinvested. The crucial difference between monetisation by means of debt relief and the current approach of the PSPP and PEPP programmes is that sovereign debt continues to exist and remains visible in the debt-to-GDP ratios. The European Fiscal Compact and the debt brake mean that a "red line" thus remains in effect, both in legal terms and with respect to the capital market.

The monetisation of sovereign debt involves the risk of a renewed increase in debt-to-GDP ratios. Particularly in the euro area, which consists of countries in different financial positions, this could cause conflicts that could establish monetisation as a permanent tool. A perpetuation that is based on the granting of debt relief at the moment when sovereign debt is purchased and involves the collusive behaviour of the Governing Council's majority and member

16 What is more, as the profits of the NCBs are expected to be lower in the future, the individual write-off process may take a very long time. As an example, for the year 2020, the Bundesbank did not distribute a profit to the Federal Government for the first time since 1979. The Bundesbank's balanced annual result was mainly due to a greater level of risk provisioning (provisions for general risks were raised by €2.4 billion), see Deutsche Bundesbank (2021a). Meanwhile, owing to negative interest rates, the Bundesbank's PSPP and PEPP public sector portfolios generated negative interest income, see Deutsche Bundesbank (2021b, 69-70).

17 According to De Grauwe (2021), already the purchase of government bonds by a central bank is "equivalent to debt relief granted to the government" because "the government does not have to pay interest any longer on its outstanding bonds held by the central bank".

18 In the event of inflationary tendencies, the ESCB would have to absorb the liquidity created through the government bond purchases in another way or curb money creation by commercial banks, e.g. by selling other assets, raising the key interest rates, or increasing reserve requirements. As an alternative, the ECB could issue its own debt certificates as liquidity-absorbing operations, see European Central Bank (2014), Article 9(1) and Article 13. An even more unconventional approach would be to introduce a reserve requirement for lending operations. See Troost and Hersel (2013, 14-16).

19 The sale of larger government bond holdings would cause the rates of these bonds to drop; the value of these holdings would thus have to be adjusted accordingly in the balance sheets of the financial sector. On the other hand, rising effective interest rates would make the issue of new government debt more costly, in particular for crisis-ridden countries.

Table 1

**Divergence of PSPP and PEPP cumulative net purchases of public sector securities from the ECB's capital key (as of 31 May 2021)**

| Countries | Cumulative net purchases of PSPP and PEPP[1] (billion euros) | Share of cumulative net purchases of PSPP and PEPP (%) | Relative ECB capital key[2] (%) | Divergence of PSPP and PEPP cumulative net purchases from relative capital key (percentage points) | Divergence of PSPP and PEPP cumulative net purchases from relative capital key (%) | Divergence of PSPP and PEPP cumulative net purchases from relative capital key (billion euros) |
|---|---|---|---|---|---|---|
| Austria | 99.90 | 3.07 | 2.93 | 0.14 | 4.93 | 4.69 |
| Belgium | 125.77 | 3.87 | 3.64 | 0.22 | 6.12 | 7.26 |
| Cyprus | 5.73 | 0.18 | 0.22 | -0.04 | -18.11 | -1.27 |
| Estonia | 0.64 | 0.02 | 0.28 | -0.26 | -92.99 | -8.52 |
| Finland | 55.06 | 1.69 | 1.84 | -0.14 | -7.85 | -4.69 |
| France | 681.47 | 20.95 | 20.42 | 0.53 | 2.57 | 17.10 |
| Germany | 866.58 | 26.64 | 26.36 | 0.28 | 1.06 | 9.09 |
| Greece | 25.68 | 0.79 | 2.47 | -1.68 | -68.08 | -54.78 |
| Ireland | 55.89 | 1.72 | 1.69 | 0.02 | 1.47 | 0.81 |
| Italy | 608.30 | 18.70 | 16.99 | 1.71 | 10.08 | 55.69 |
| Latvia | 4.22 | 0.13 | 0.39 | -0.26 | -66.70 | -8.45 |
| Lithuania | 7.43 | 0.23 | 0.58 | -0.35 | -60.53 | -11.40 |
| Luxembourg | 5.09 | 0.16 | 0.33 | -0.17 | -52.54 | -5.63 |
| Malta | 1.56 | 0.05 | 0.10 | -0.06 | -54.22 | -1.85 |
| Netherlands | 181.48 | 5.58 | 5.86 | -0.28 | -4.80 | -9.15 |
| Portugal | 70.23 | 2.16 | 2.34 | -0.18 | -7.76 | -5.91 |
| Slovakia | 22.10 | 0.68 | 1.15 | -0.47 | -40.68 | -15.15 |
| Slovenia | 14.09 | 0.43 | 0.48 | -0.05 | -10.03 | -1.57 |
| Spain | 421.60 | 12.96 | 11.92 | 1.04 | 8.69 | 33.72 |

Notes: [1] Cumulative net purchases of public sector securities under the PSPP and PEPP – excluding purchases of supranational issuers, which, however, can also be used indirectly for government financing (ESM bonds). [2] Relative capital key (valid since 1 February 2020) of the Eurosystem members.

Source: ECB information on the capital key (https://www.ecb.europa.eu/ecb/orga/capital/html/index.en.html), ECB data on the PSPP programme (https://www.ecb.europa.eu/mopo/implement/omt/html/index.en.html#pspp) and on the PEPP programme (https://www.ecb.europa.eu/mopo/implement/pepp/html/index.en.html), own calculations.

states, would make the ESCB into a money printing press in the traditional sense and in accordance with Modern Monetary Theory (MMT).

## Scenario analysis

In the following, we look at two alternative debt relief scenarios that differ considerably in terms of procedure and volume. The approach outlined in this article involving ESCB write-off bonds and a relief agreement issued by an EU debt agency is based on the government bonds in the ESCB portfolio. The holdings purchased within the scope of the PSPP and PEPP programmes are published by the ECB.[20] Since some of these, however, deviate considerably from the ECB's capital key, the actual portfolio allocation according to issuing member states should be chosen as the existing basis for ESCB debt relief. For example, too many government bonds – in terms of the ECB capital key – have been bought from Italy (a difference of 10.1%, equivalent to €55.7 billion), Spain (8.7%, €33.7 billion) and France (2.6%, €17.1 billion), while too few have been purchased from the Netherlands (minus 4.8%, minus €9.1 billion), see Table 1.

The considerable negative differences for some smaller and less indebted countries shown in Table 1, for one thing, are due to the limited availability of purchasable securities. In addition, a low degree of creditworthiness prevented PSPP purchases of Greek and temporarily of Cypriot government bonds. As a result, these countries initially only have limited scope for relief.

Regarding these available government bond portfolios held by the central banks, two debt relief scenarios are considered here:

20 By contrast, there is no transparency about purchases of government bonds as part of the Agreement on Net Financial Assets (Hansen and Meyer, 2020c, 232-233).

Table 2

**Effects of debt relief of €795.906 billion as per ECB capital key (forecasts as of 12 May 2021)**

| Countries | ECB capital key[1] (%) | Debt relief in accordance with ECB capital key (billion euros) | Debt-to-GDP ratio as of 31 December 2021[2] (%) | Debt-to-GDP ratio as of 31 December 2021 after debt relief[2] (%) | Reduction of debt-to-GDP ratio with debt relief (%) | Cumulative budget deficits in 2020-2022[3] (billion euros) | Coverage of 2020-2022 budget deficits with debt relief (%) |
|---|---|---|---|---|---|---|---|
| Austria | 2.38 | 18.95 | 87.19 | 82.38 | -5.52 | 75.40 | 25.13 |
| Belgium | 2.96 | 23.58 | 115.33 | 110.41 | -4.27 | 103.80 | 22.72 |
| Cyprus | 0.18 | 1.39 | 112.27 | 105.94 | -5.64 | 2.80 | 49.74 |
| Estonia[4] | 0.23 | 1.82 | 21.48 | 15.06 | -29.89 | 3.90 | 46.75 |
| Finland | 1.49 | 11.89 | 71.03 | 66.24 | -6.74 | 29.80 | 39.90 |
| France | 16.61 | 132.21 | 117.42 | 111.98 | -4.63 | 539.10 | 24.52 |
| Germany | 21.44 | 170.64 | 73.02 | 68.15 | -6.67 | 495.70 | 34.42 |
| Greece | 2.01 | 16.01 | 208.83 | 199.53 | -4.45 | 39.30 | 40.74 |
| Ireland | 1.38 | 10.96 | 61.37 | 58.56 | -4.59 | 49.50 | 22.14 |
| Italy | 13.82 | 109.97 | 159.82 | 153.48 | -3.97 | 465.20 | 23.64 |
| Latvia | 0.32 | 2.52 | 47.42 | 39.28 | -17.16 | 4.30 | 58.66 |
| Lithuania | 0.47 | 3.75 | 51.95 | 44.64 | -14.08 | 11.10 | 33.75 |
| Luxembourg | 0.27 | 2.13 | 27.01 | 23.89 | -11.53 | 2.90 | 73.53 |
| Malta | 0.09 | 0.68 | 64.71 | 59.71 | -7.71 | 3.70 | 18.35 |
| Netherlands | 4.77 | 37.93 | 57.95 | 53.39 | -7.86 | 91.20 | 41.59 |
| Portugal | 1.90 | 15.15 | 127.24 | 120.14 | -5.58 | 29.20 | 51.88 |
| Slovakia | 0.93 | 7.41 | 59.44 | 51.79 | -12.87 | 16.10 | 46.04 |
| Slovenia | 0.39 | 3.12 | 79.02 | 72.67 | -8.03 | 10.50 | 29.68 |
| Spain | 9.70 | 77.19 | 119.56 | 113.12 | -5.38 | 281.60 | 27.41 |
| EU19 (euro area) | 81.33 | 647.30 | 102.35 | 96.94 | -5.29 | 2254.90 | 28.71 |

Notes: [1] Shares of Eurosystem member countries in ECB capital (capital key effective since 1 February 2020). [2] Own calculations based on EU Commission forecasts on debt and GDP. [3] Forecasts of the EU Commission. [4] Debt relief exceeds the NCB's current holdings of government bonds purchased as part of PSPP and PEPP.

Source: ECB information on the capital key (https://www.ecb.europa.eu/ecb/orga/capital/html/index.de.html). Own calculations based on the AMECO annual macro-economic database of the European Commission's Directorate General for Economic and Financial Affairs. Spring 2021 Economic Forecast as of 12 May 2021.

- scenario (i): debt relief based directly on the credit volume of the €750 billion NGEU recovery fund
- scenario (ii): debt relief of 60% of the pre-crisis 2019 GDP, based on the European Fiscal Compact and the Maastricht Treaty.

The focus is on the effects on the debt-to-GDP ratios and the potential coverage of the budget deficits of the euro area member states. Our calculations assume *ceteris paribus* and are based on data and forecasts of the European Commission.[21]

## Debt relief as a substitute for the NGEU recovery fund

As a possible alternative to the recovery fund, scenario (i) involves debt relief totalling €795.906 billion for all EU member states. This amount results from the power of the Commission "to borrow funds on capital markets on behalf of the Union up to €750 billion in 2018 prices" (Council of the European Union, 2020, Article 5(1)).[22] This approach is based on the assumption that debt relief will be conducted in 2021, i.e. when payments from NGEU begin. With debt relief, the entire budget would be transferred to the countries at that time. In contrast to NGEU, there would be no implicit transfers through disproportionate access to grants and loans by the euro area member countries because distribution is carried out in accord-

21 With regard to the following scenarios (i) and (ii), it should be noted that by using some working assumptions, the budgetary and economic impacts of the NGEU recovery instrument were already incorporated into the European Commission's forecasts (European Commission, 2021b, 41-42).

22 Debt relief volume as an NGEU substitute is calculated on the basis of an annual deflator of 2% as 750 * $(1.02)^3$ = €795.906 billion for the year 2021.

Figure 2
**Effects of debt relief as per ECB capital key on debt-to-GDP ratios (as of 31 December 2021)**

Note: Debt relief volume of €795.906 billion.

Figure 3
**Coverage of cumulative budget deficits in 2020-2022 by means of debt relief as per ECB capital key**

Note: Debt relief volume of €795.906 billion.

ance with the ECB's capital key. For the euro area, this results in a volume of €647.3 billion (see Table 2). Measured by past debt levels, some small countries such as the Baltic states would benefit from this. These countries have lower debt-to-GDP ratios anyway, some well under 60% (see Figure 2). However, the reduction effect is much lower for the heavily indebted countries Greece (the debt-to-GDP ratio drops from 208.8% to 199.5%), Italy (from 159.8% to 153.5%) and Portugal (from 127.2% to 120.1%). As for the euro area as a whole, debt relief would reduce the share of government debt in relation to GDP from 102.4% to 96.9%.

The goal of considerably reducing debt levels is therefore not achieved, in particular for those euro area countries whose debt sustainability is acutely compromised. However, as shown in Figure 3, debt relief would considerably cover the cumulative budget deficits of the years 2020 to 2022 in Portugal (51.9%) and Greece (40.7%). For Spain (27.4%), France (24.5%) and Italy (23.6%), deficit coverage would be a great deal lower. More than one quarter of the cumulative budget deficits 2020-2022 of the euro area as a whole would be covered. The portfolio of Estonian government bonds held by the ESCB, however, would be too small (by approximately €1.2 billion) for the implementation of debt relief in accordance with scenario (i).[23] In order to provide equal treatment, the missing bonds would have to be bought immediately or gradually on the secondary market. This could, however, coincide with a distortion of risk premiums and even additional capacity for debt.[24]

## Debt relief referring to the European Fiscal Compact and the Maastricht Treaty

Scenario (ii) is an extreme theoretical scenario. In contrast to scenario (i), there is no absolute target figure for debt relief. Instead, relief is measured for all countries by GDP

23 If a decision were made to conduct debt relief only through the holdings of the NCBs, these figures would likely increase somewhat because eight-ninths of the purchases are conducted by the NCBs and one-ninth by the ECB. On the allocation of portfolios of PSPP and PEPP, see Article 6 of Decision (EU) 2020/188 of the ECB as well as Article 1(2)(a) and Article 5 of Decision (EU) 2020/440 of the ECB.

24 The government bonds still needed for debt relief are likely to continue decreasing due to further PSPP and PEPP purchases. The calculations used in this analysis are based on holdings as of 31 May 2021.

Table 3

**Effects of debt relief of 60% of the 2019 GDP (forecasts as of 12 May 2021)**

| Countries | Debt relief to the amount of 60% of the 2019 GDP[1] (billion euros) | Debt-to-GDP ratio as of 31 December 2021[2] (%) | Debt-to-GDP ratio as of 31 December 2021 after debt relief[2] (%) | Reduction of debt-to-GDP ratio with debt relief (%) | Cumulative budget deficits in 2020-2022[3] (billion euros) | Coverage of 2020-2022 budget deficits with debt relief (%) |
|---|---|---|---|---|---|---|
| Austria[4] | 238.56 | 87.19 | 26.57 | -69.53 | 75.40 | 316.39 |
| Belgium[4] | 285.78 | 115.33 | 55.71 | -51.70 | 103.80 | 275.32 |
| Cyprus[4] | 13.38 | 112.27 | 51.45 | -54.17 | 2.80 | 477.86 |
| Estonia[4] | 16.86 | 21.48 | -37.89 | -276.39 | 3.90 | 432.31 |
| Finland[4] | 144.18 | 71.03 | 12.94 | -81.78 | 29.80 | 483.83 |
| France[4] | 1462.56 | 117.42 | 57.29 | -51.21 | 539.10 | 271.30 |
| Germany[4] | 2069.46 | 73.02 | 13.96 | -80.88 | 495.70 | 417.48 |
| Greece[4] | 110.04 | 208.83 | 144.89 | -30.62 | 39.30 | 280.00 |
| Ireland[4] | 213.66 | 61.37 | 6.46 | -89.47 | 49.50 | 431.64 |
| Italy[4] | 1074.54 | 159.82 | 97.89 | -38.75 | 465.20 | 230.98 |
| Latvia[4] | 18.24 | 47.42 | -11.42 | -124.08 | 4.30 | 424.19 |
| Lithuania[4] | 29.28 | 51.95 | -5.23 | -110.08 | 11.10 | 263.78 |
| Luxembourg[4] | 38.10 | 27.01 | -28.61 | -205.95 | 2.90 | 1313.79 |
| Malta[4] | 8.16 | 64.71 | 4.71 | -92.73 | 3.70 | 220.54 |
| Netherlands[4] | 486.12 | 57.95 | -0.40 | -100.69 | 91.20 | 533.03 |
| Portugal[4] | 128.34 | 127.24 | 67.07 | -47.29 | 29.20 | 439.52 |
| Slovakia[4] | 56.34 | 59.44 | 1.30 | -97.81 | 16.10 | 349.94 |
| Slovenia[4] | 29.04 | 79.02 | 19.88 | -74.85 | 10.50 | 276.57 |
| Spain[4] | 746.88 | 119.56 | 57.29 | -52.08 | 281.60 | 265.23 |
| EU19 (euro area) | 7169.52 | 102.35 | 42.41 | -58.56 | 2254.90 | 317.95 |

Notes: [1] Own calculations based on EU Commission data. [2] Own calculations based on EU Commission forecasts on debt and GDP. [3] Forecasts of the EU Commission. [4] Debt relief exceeds the NCB's current holdings of government bonds purchased as part of PSPP and PEPP.

for 2019, i.e. the year before the onset of the coronavirus crisis.[25] In accordance with the European Fiscal Compact, a debt-to-GDP ratio of 60% is a reference value that member states should not exceed.[26] Based on this, debt relief of 60% of the 2019 GDP is analysed in this scenario. Totalling €7,169.5 billion for the euro area, this debt relief, as expected, results in a considerable improvement in the debt-to-GDP ratios (see Table 3). After debt relief in 2021, Greece is the only country, out of seven previously, to have government debt that exceeds its GDP. The Greek debt-to-GDP ratio falls from 208.8% to 144.9%. The previously problematic levels in Italy (from 159.8% to 97.9%) and Portugal (from 127.2% to 67.1%) would be reduced substantially. All other countries would have a debt-to-GDP ratio of less than 60% after debt relief, including Spain (from 119.6% to 57.3%), France (from 117.4% to 57.3%) and Germany (from 73.0% to 14.0%). For the euro area as a whole, such debt relief reduces the debt ratio from 102.4% to 42.4% and the cumulative budget deficits 2020-2022 would be covered about threefold (see Figure 5). Since the financial burdens resulting from the coronavirus crisis will not be over soon, countries will welcome this surplus coverage.

However, it would be highly problematic that, for reasons of equal treatment, it would be necessary to grant five countries what, in some cases, would be a considerable additional debt margin (e.g. €19.6 billion for Luxembourg and €10.8 billion for Estonia), as the debt relief exceeds the debt of these countries (see Table 3 and Figure 4). In

25 By using the pre-crisis GDP as a reference value, one can avoid giving unfavourable treatment to those countries particularly affected by the crisis. See also Stelter (2020, 153-160).

26 See Article 4 of the Treaty on Stability, Coordination and Governance in the Economic and Monetary Union (2012).

Figure 4
**Effects of debt relief of 60% of the 2019 GDP on debt-to-GDP ratios (as of 31 December 2021)**

Figure 5
**Coverage of cumulative budget deficits in 2020-2022 by means of debt relief of 60% of the 2019 GDP**

addition, the holdings of government bonds in the portfolios of the central banks do not suffice to implement the designated debt relief for any country. In terms of figures, there is a lack in particular of German (€1,202.9 billion), French (€781.1 billion) and Italian (€466.2 billion) securities. The adverse incentives involved in eliminating this lack of securities – which is considerably more common and more pronounced than in scenario (i) – are likely to be prohibitively high.

## Conclusions

In the wake of the coronavirus crisis, there has been a considerable rise in the debt-to-GDP ratios of euro area countries. Without external help such as the PEPP government bond purchases of the ESCB, some southern European countries in particular face the risk of losing access to the capital market. This article thus seeks to find an approach that gives the countries more own scope of action and that accords with EU treaties but that does not – unlike the NGEU recovery fund – introduce redistribution elements and shift powers to the EU level. To this end, the article analyses debt relief by the ESCB which is conducted through an EU debt agency on the basis of government bonds purchased by the ESCB under the PSPP and PEPP programmes.

Despite its immediate effect, this form of debt relief would prevent negative equity at the central banks, but there would be major legal hurdles to consider. A temporary exception would require legislative authorisation because the prohibition of monetary financing (Article 123 TFEU) is a key rule of EU economic law. In addition, the conducted scenario analysis has identified trade-offs with regard to practical implementation. Debt relief based on the recovery fund volume (€750 billion in 2018 prices) and allocated in accordance with ECB capital shares would by and large be neutral from a distributional point of view, but it would hardly bring on more sustainable debt ratios. In contrast, debt relief of 60% of the pre-crisis 2019 GDP, which is based on the European Fiscal Compact, would significantly reduce debt ratios, but this would also give many countries considerable extra debt capacity. The result is a trilemma consisting of the wish to ensure equal treatment, significant debt relief, and avoidance of adverse incentives. The latter is impeded in particular by the fact that, as the scope of the relief increases, it would be necessary to continue replenishing the government bonds held by the ESCB.

There is also the unanswered question as to how, in the event of inflationary tendencies, the additional liquidity resulting from the PSPP and PEPP purchases can be reabsorbed. Improved conditions could quickly lead to an increase in aggregate demand and bank lending. In addition, the credible non-recurring nature of such debt relief would be decisive for the future reputation and independence of the central banks involved. National consolidation efforts after the relief are also likely to be of key importance. If, however, the use of an ESCB debt relief as a last resort brings along a risk of the permanent monetisation of government debts, inflation and capital flight could eventually lead to a dead end.
